# Effect of Piperine on Saltiness Perception

**DOI:** 10.3390/foods12020296

**Published:** 2023-01-08

**Authors:** Rachael Moss, Cassie Fisher, Mackenzie Gorman, Sophie Knowles, Jeanne LeBlanc, Christopher Ritchie, Kaelyn Schindell, Laurel Ettinger, Matthew B. McSweeney

**Affiliations:** School of Nutrition and Dietetics, Acadia University, Wolfville, NS B4P 2K5, Canada

**Keywords:** threshold testing, cross-modal interactions, salt reduction, chemical irritants, chemesthesis

## Abstract

Chemical irritants, like piperine, have the potential to increase human perception of tastes and odours, including saltiness. This cross-modal interaction could help the food industry develop new salt-reduced food products that maintain their salty taste. The objective of this study was: firstly, to determine the detection threshold of piperine (*n* = 72), secondly to evaluate piperine’s influence on saltiness perception in model solutions (*n* = 78), and lastly to identify piperine’s effect on sensory perception of low sodium soup using temporal check-all-that-apply (TCATA; *n* = 75). The group mean of the individual threshold was 0.55 ± 0.15 ppm. Piperine increased the saltiness perception of the model solutions, but it also increased the bitterness and decreased the sweetness of the solutions. The piperine significantly increased the saltiness intensity of the soups (evaluated using a generalized labelled magnitude), but during the TCATA task, the salty attribute was selected less for the soup with piperine than the control (based on the average proportion of selection). The TCATA indicated that the peppery attribute dominated the participants’ perception of the soup with piperine. More studies are needed to assess piperine’s cross-modal interactions.

## 1. Introduction

World-wide, salt is consumed in quantities well above the 5 g/d (2 g/d of sodium) recommended by the World Health Organization (WHO) [1]. Furthermore, excessive salt intake has been directly associated with negative health outcomes [2,3,4,5]. In Canada, increased salt intake is obtained primarily from packaged foods and restaurant foods rather than added salt during cooking or at the table, which accounts for only 11% of sodium intake [2]. Reducing salt intake at a population level has become a primary challenge in mitigating salt consumption-related disease, and is a key recommendation by health agencies globally [2,6,7,8,9]. Moreover, the WHO has proposed salt reduction as a the key dietary target for 2025 to reduce mortality from many diseases (high blood pressure, heart disease, and stroke) related to high salt intake [2,9].

Many food companies have also begun reducing the salt content of their products, however, concerns around the effects of salt reduction exist [4,5]. Salt has many key roles in food products [4,5,10]. Reducing the salt content of foods, while maintaining physiochemical properties, product stability, and desired sensory attributes is a significant challenge for the food industry [4,5,10]. Reducing salt has also been shown to impact a food products’ sensory appeal and flavour complexity, which can negatively impact consumer acceptance [4]. Flavour is considered the primary driver for both consumer food choice and food intake [11]. Therefore, altering flavour with alternative methods or ingredients may be the solution to meeting consumer food preferences while also supporting salt reduction and positive health outcomes.

Flavour is defined as the combination of gustation (taste), olfaction (odour), and trigeminal sensations [12,13]. Trigeminal sensations or chemesthesis, occur when chemical irritants stimulate the trigeminal nerve in the oral cavity [11,14]. Trigeminal sensations are part of the somatosensory system and are integral to the mammalian pain and warning system [15]. Unlike tastants and odourants, which interact with the gustatory receptors or olfactory receptors, chemical irritants interact with temperature, pain, and touch receptors in the oral region [16,17]. This interaction leads to sensations such as burning, warmth, heat, cooling, and fizziness [11]. Cross-modal and sensation transference research has demonstrated that consumers associate temperature with other sensory characteristics [18]. There are many foods containing compounds that act as chemical irritants, which have been shown to affect consumer flavour perception similarly [19,20,21,22].

Different oral chemical irritants act by producing different sensations, including tickling, pungency, burning, stinging, or cooling [23]. The burn of hot peppers and mustards, the coolness of peppermint, and the tingle of carbonated beverages are all trigeminal sensations that occur, as a result of chemical irritants [24]. Capsaicin, for example, is the main chemical irritant found in chili peppers and is one of the most studied oral chemical irritants [25,26]. Capsaicin has been shown to elicit trigeminal sensations which results in an increased saltiness perception in humans when added to a sodium chloride (NaCl) solution, even at reduced concentrations [26,27]. However, capsaicin may not be an acceptable ingredient to add to certain foods, as it may impart undesirable sensory properties [15]. Capsaicin’s spicy taste is polarizing to consumers, especially those living in the Western world [15]. This indicates that other chemical irritants need to be explored to determine whether they too can increase saltiness perception in consumers. 

Piperine (a compound found in black pepper) is another chemical irritant and an alternative to capsaicin. Black pepper (*Piper nigrum*) is a widely used spice known for its distinct biting and stinging characteristic that is attributed to the chemical irritant, piperine [20,21]. Piperine has been suggested to have several health benefits, as well as flavour-enhancement properties [20,21,28]. There has been some indication that piperine may also enhance saltiness perception; however, this is unclear due to limited research and opposing findings [29,30], thereby suggesting a need for new research. Studies have looked at the use of oral rinses with chemical irritants such as capsaicin and piperine and their effects on perceived sweetness, bitterness, sourness, and saltiness [14,25,29,30,31]. However, there remain significant knowledge gaps related to how different chemical irritants affect human perception of various flavours, especially within a food matrix. Some research has suggested that chemical irritants interfere with taste and odour [24,25,32], while other studies have suggested chemical irritants increase the perception of taste and odours [17,33,34,35]. Therefore, there is a need to evaluate alternative chemical irritants to determine whether they affect consumer perception of taste and odour, specifically saltiness.

This study aims to determine the impact of piperine (black pepper) on consumers’ perception of saltiness. It has three specific objectives: 1. To determine the detection threshold of piperine; 2. To identify the cross-modal interactions of piperine in model solutions (salt and a hydrocolloid); 3. To determine if the inclusion of piperine in a sodium-reduced food item increases saltiness perception.

## 2. Materials and Methods

### 2.1. Participants

This study was approved by the Acadia University Research Ethics Board (13-72). Participants were recruited from the local community surrounding Acadia University (Wolfville, Nova Scotia, Canada). Participants were screened before participating in the three different trials (which will be referred to as threshold testing, model solutions, and soup). Eligibility criteria (adapted from Hayes et al. [36]) included participants who were between 18–65 years old, not breastfeeding or pregnant, had no known defects of smell or taste, had not smoked in 30 days, and had no difficulty swallowing. The study also took place during the COVID-19 pandemic; therefore, participants were asked about their health status, potential exposure to the virus, loss of smell and taste, and if they had recently (within two months) tested positive for the COVID-19 virus. All participants gave written informed consent. Additionally, all trials listed below were recruited separately, but participants were not excluded if they had been involved in a previous trial (Participant characteristics can be seen in Table 1). All trials took place in white individual sensory booths, with a controlled temperature and ambient lighting. The questionnaires were completed on computers using the Compusense Cloud software (Guelph, Ontario, Canada).

### 2.2. Threshold Testing

The threshold testing followed a method adapted from Nolden et al. [37]. A stock solution of 9120 ppm piperine was prepared following the method in Nolden et al. [37]. Six concentrations of piperine were evaluated using the whole sip and spit method. The six concentrations were 0.14 ppm, 0.28 ppm, 0.57 ppm, 1.14 ppm, 2.28 ppm, and 4.56 ppm in reverse osmosis water. Detection thresholds were collected using the 3-alternative forced choice method based on the ASTM method E-679 [37]. The participants (*n* = 72) were asked to swish the sample (10 mL at 25 °C) in their mouth for three seconds and then spit out the sample [37]. The participants were instructed to take a one-minute break between set of samples and cleanse their palate with reverse osmosis water. After spitting out the sample, they waited five seconds before evaluating the next sample. After they had tasted all three samples, they were asked to identify which sample was different. 

### 2.3. Model Solutions

The piperine at the detection threshold (based on the threshold testing above) was incorporated into solutions of 0.2% xanthan gum [38], a solution with 0.75 mM NaCl [27], and a solution with both 0.2% xanthan gum and 0.75 mM NaCl. In addition, a solution with piperine (at detection threshold), 0.2% xanthan gum, and 0.75 mM NaCl was included. All the solutions were prepared in reserve osmosis water. The samples (15 mL at 25 °C) were presented to participants (*n* = 78) following a completely randomized design in clear cups and were blinded with random three-digit codes. The participants were instructed to take a one-minute break between samples to cleanse their palate with reverse osmosis water. The participants used a general labelled magnitude scale [39] to evaluate the saltiness, bitterness, sweetness, sourness, and umami (savoury) intensity of each of the solutions, as well as the intensity of the burning and stinging sensation.

### 2.4. Soup

A commercially available soup (ingredients: chicken broth (water, chicken stock), salt, yeast extract (barley), natural flavour, sugars (dextrose), canola or soybean oil.) labelled as low sodium, was purchased from a local grocery store. The manufacturer’s instructions were followed to prepare the two batches of soup. The soup was homogenous and did not include large pieces of vegetables or meat. After preparation, piperine at the detection threshold (based on the threshold testing above) was added to one batch of the soup (referred to as soup with piperine) and agitated with a wire whisk for 90 s. The other batch was not modified (referred to as control). The samples were presented in clear cups with lids and a 10 mL spoon was provided for each evaluation. The samples were blinded with three-digit codes and presented at 60 °C. 

The samples (30 mL) were evaluated by the participants (*n* = 75) for their overall liking and liking of appearance, flavour, and texture on a nine-point hedonic scale (1 = Dislike Extremely and 9 = Like Extremely). They also evaluated the saltiness intensity using a general labelled magnitude scale. The participants then took a four-minute break and evaluated the samples using the temporal check-all-that-apply method [40]. Before evaluation began, a researcher explained TCATA to the participants and defined the attributes (salty, savoury, peppery, sweet, metallic, bitter, and sour) for the participants. The attributes were chosen based on a preliminary study asking 25 participants to evaluate the two soups using check-all-that-apply (CATA; data not shown). The most frequently cited attributes in the CATA were included in the TCATA task. A demonstration of TCATA was conducted and all questions from the participants were answered. Furthermore, instructions were presented by the software. Participants were asked to place the sample (60 mL at 60 °C) in their mouth and evaluate it for up to 60 s [41]. They were asked to take a regular to full mouthful and press the “Start” button on the computer screen simultaneously. Participants were then asked to select the attribute that describes the sample. Each selected term faded after 5 s, and the participants had to re-select the term if it still applied. The participants were instructed to click the attributes after swallowing the sample as well. If the participants were not perceiving any of the attributes, they were asked to stop their evaluation by clicking the stop button. The order of attributes and the presentation orders for each sample were balanced among the participants [41]. 

### 2.5. Statistical Analysis

#### 2.5.1. Threshold Testing

The analysis of the threshold testing followed the methods outlined by Nolden et al. [37] and Lawless and Heymann [42]. Briefly, the individual detection thresholds were calculated as the geometric mean between the first correct answer followed by all correct answers. The lowest concentration was selected. Group detection thresholds were then calculated as the geometric mean of all individual detection thresholds. 

#### 2.5.2. Model Solutions

The intensity of saltiness, bitterness, sweetness, sourness, umami (savoury), and burning and stinging sensation were evaluated using a general labelled magnitude scale [39] and were assessed using a two-way analysis of variance (ANOVA) and Tukey’s Honest Significant Difference (HSD) test. 

#### 2.5.3. Soup

The results of the hedonic scales and general labelled magnitude scales were compared using paired *t*-tests. The results of the TCATA were analyzed according to Castura et al. [40]. Aggregated data across all participants were described using line plots as the consumer citation proportion was smoothed using a cubic smoothing spline and plotted 0.1-s increments for each sample. An analysis of the average proportion was conducted [43]. All analyses were completed using XLSTAT software (Version 2022.1.2, New York, NY, USA) in Microsoft Excel^TM^.

## 3. Results and Discussion

### 3.1. Threshold Testing and Model Solutions

The group mean of the individual threshold was 0.55 ± 0.15 ppm. This result is very similar to that of Nolden et al. [37] who identified that the group geometric mean was 0.58 ± 0.25 ppm for piperine for American participants.

The piperine (at detection threshold) was then added to different model solutions including 0.2% xanthan gum and 0.75 mM NaCl to evaluate piperine’s cross-modal interactions. The results can be found in Table 2. No significant differences were found in the solutions in terms of sourness and umami (*p* > 0.05). However, significant differences were found in the intensity of the sweetness, saltiness, and bitterness of the solutions (*p* < 0.05). The 0.2% xanthan gum solution was found to be significantly sweeter than the other solutions (*p* < 0.05). Xanthan gum has been found to decrease sourness and bitterness [44], which may explain why the sweetness of the 0.2% xanthan gum solution was scored higher by participants. Furthermore, xanthan gum has been found to increase the sweetness of yogurts [45]. Additionally, sourness and bitterness have a suppressive effect on sweetness [46] and this could explain why the 0.2% xanthan gum solution was significantly sweeter, as it did not contain piperine (adding bitterness). This result may also be due to a contrast effect, as all other samples contained either piperine (leads to bitterness and burning intensity [47]) or sodium chloride (saltiness). Although the participants were instructed to take a one-minute break between samples, a longer break between samples may be necessary to decrease contrast effects in future studies.

Bitterness intensity was also significantly higher in the solutions that contained piperine solution (except the 0.55 ppm piperine and 0.75 mM NaCl) compared to the 0.2% xanthan gum (*p* < 0.05). Once again, this may be due to piperine contributing a bitter taste, [47,48]. This result may also be explained by perceptual interaction as Lim and Green [49] identified that bitterness and a burning or stinging sensation are qualitatively similar. Furthermore, the bitterness of the 0.55 ppm piperine and 0.2% xanthan gum solution, although not significantly different than the 0.55 ppm piperine solution, was reduced. The sodium chloride, however, did not have a significant effect on the bitterness intensity of the piperine (*p* > 0.05).

Sodium chloride also did not significantly affect the burning or stinging sensation caused by the piperine (*p* > 0.05). However, the 0.2% xanthan gum significantly decreased the burning or stinging sensation from the piperine (*p* < 0.05). This result was found by Nasrawi and Pangborn [38] when they evaluated 0.2% xanthan gum and capsaicin. They found that the xanthan gum reduced the magnitude of the burning sensation, but not the duration of the burn. This study only investigated the intensity of the burning or stinging sensation. Future studies should investigate the effect of xanthan gum and other hydrocolloids on the duration of the burning or stinging sensation from piperine. Additionally, capsaicin has been found to increase the thickness discrimination threshold [50], but this was not investigated for piperine in this study. Lastly, the piperine did significantly increase the saltiness perception of the solutions. The 0.55 ppm piperine and 0.75 mM NaCl solution was perceived to be significantly higher than the 0.75 mM NaCl solution (*p* < 0.05). This result agrees with past studies demonstrating that chemical irritants (in this study piperine) can increase the perception of tastants and odourants [11]. Capsaicin serves to increase sensitivity to other tastants [31,33,34], specifically salt [27], which may also be true for piperine as the saltiness perception increased in this study. More studies need to be conducted investigating the cross-modal interactions of piperine as limited literature exists. In addition, more investigation needs to be completed on the inclusion of piperine in different foodstuffs. As such, the following section investigates the addition of piperine to low-sodium soup. 

### 3.2. Soup

Mean liking scores were computed for consumers’ liking of appearance, flavour, and texture, as well as overall liking of low-sodium soup with and without piperine. Results are shown in Table 3. Overall, the appearance of the piperine soup was liked significantly more than the control soup (*p* < 0.05). However, the flavour of the control soup was liked by consumers more than the piperine soup (*p* < 0.05). Minimal differences were found for consumer liking of texture for both soups. Saltiness intensity was perceived as significantly higher in the piperine soup (*p* < 0.05), which agrees with the results of the model solutions (Table 2). Overall liking was significantly higher for the control soup compared to the piperine soup (*p* < 0.05). This result was expected as the addition of piperine is associated with bitterness and pungency [20]. Moreover, a study by Scott et al. [51] looking at the perception of tomato soup with capsaicin from chili powder found that disgust was associated with pungency from the added capsaicin. While Scott et al. [51] did not evaluate piperine, it may be a possible explanation for the differences observed in overall liking of the control and piperine soups in this study.

**Figure 1:**
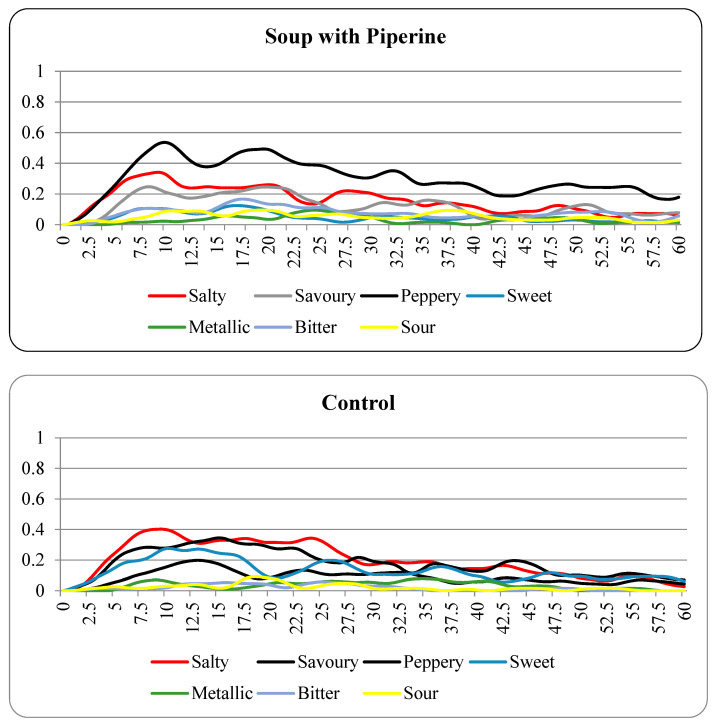
TCATA curves obtained with consumers for the control and the soup with piperine.

Figure 1 and Table 4 show TCATA attributes salty, savoury, peppery, sweet, metallic, bitter, and sour selected by participants during the evaluation of both soups. As expected, the soup with piperine had the peppery attribute selected more frequently than the control soup. The soup with piperine also demonstrated reduced sweetness in comparison to the control soup and was higher in bitter and sour attributes. The average proportion of participants’ citations for the saltiness of the control soup was higher than when the participants evaluated the soup with piperine. This result disagreed with the results of the general labelled magnitude scale (Table 3), which identified that the saltiness was significantly higher in the soup with piperine. Previous studies have also shown that oral chemical irritants added to both solutions and soup suppressed sweetness significantly and mildly suppressed saltiness, which aligns with the results of the TCATA [29,52]. Overall, the TCATA demonstrates that the intensity of salty, savoury, and sweet attributes was reduced with the addition of piperine to the low-sodium soup, while the intensity of peppery, bitter, and sour attributes was increased. This result also indicates that the use of dynamic sensory methodologies can identify differences in sensory perception that may be missed if only static scales are used.

A previous study from Lawless and Stevens [29] found similar findings when assessing oral irritation induced by rinses containing capsicum, oleoresin, and piperine. Researchers found decrements in the taste intensity of citric acid, quinine, and sucrose, although, no effects were noticed on salt [29]. Piperine as a chemical irritant was also found to have more significant decrements in perceived intensity [29]. While minimal research exists on flavour effects related to piperine and oral irritation, many studies have been conducted on capsaicin. Many of these studies have shown reduced sweetness with capsaicin and suppressed flavour intensities [25,53]. Additionally, sourness is unaffected by capsaicin and desensitization for salt and saltiness has also been demonstrated, suggesting that spice or pungency may enhance salt flavour [25,26,27,53].

Other considerations may also explain why saltiness perception was reduced in the TCATA results of this study. For example, the effects of burning intensity may vary depending on the type of chemical irritant applied. Different concentrations of piperine may also have a different effect on burn intensity or pungency and may vary depending on the matrix used. For example, a study by Eib et al. (2021) evaluated the perception of the trigeminal pungency of allyl isothiocyanate, a chemical irritant found in horseradish, and found that both the concentration of the chemical irritant as well as the composition of the food altered the perceived pungency intensity [22]. More specifically, pungency was found to be perceived longer in water compared to oil carriers [22]. This may also explain some of the findings for piperine oral irritation and perceived attributes across the different matrixes (model solutions and soup) in this study. Responses to oral irritation can also vary across different irritants as capsaicin and piperine, as both have been found to have irritation responses in different oropharyngeal regions [54]. A descriptive analysis study on oral pungency by Cliff and Heymann (1992) demonstrated that piperine and capsaicin both had pungency of primarily burning, however, they were found to have different temporal and spatial responses [55]. These factors may also explain the results obtained from this study and should be considered in future studies. The TCATA task itself may have been improved by asking the participants to complete attribute generation and then reach a consensus on the definitions of the attributes, as they would have a better understanding of the attributes included in the TCATA. In addition, this trial was completed without blocking the retronasal airway and this could have influenced the results as the aroma can perceptually influence other sensory modalities [14]. Future studies should ask participants to evaluate the tastants of different products with piperine while restricting their retronasal perception.

### 3.3. Future Studies

This study began the exploration into piperine’s cross-modal flavour interactions in both model solutions and food items. Future studies should continue to investigate how different components of foods affect the burning and stinging sensation of piperine (intensity and duration), as well as how piperine impacts tastants and odourants. Future studies should also investigate taster status (6-n-propylthiouracil [56]) and how the cross-modal interactions of piperine affect participants of different cultural backgrounds. Additionally, this study focused on how saltiness perception was affected by piperine, but future studies should also explore the impacts of piperine’s burning and stinging sensation on odour and textural perception. Differences may be present amongst consumers who regularly consume black pepper compared to those who do not, as previous studies looking at oral irritation of capsaicin have found lower perceived oral irritation or burn intensity in those who frequently consumed chili peppers [29,51,57]. Additionally, future studies should investigate if consumers who eat more spicy foods have a different response than non-consumers of spicy food to the foods containing piperine. Therefore, while limited research exists on piperine oral intensity, similar effects may be present and should be assessed in future studies with piperine. Overall, additional studies need to continue to investigate the cross-modal interactions of piperine and other chemical irritants and future studies should investigate if consumers’ detection threshold for saltiness changes with piperine addition.

## 4. Conclusions

The addition of piperine to model solutions significantly increased the participants’ saltiness perception, however, this result was not found when piperine was added to low-sodium soup and evaluated using TCATA. Further, the addition of xanthan gum decreased the burning and stinginess sensation of the piperine. The addition of piperine increased the bitterness perception in the model solutions and this interaction should be further explored in future studies. Piperine also increased the bitterness and sourness of the soup, while decreasing the sweetness and consumer acceptability. These results highlight that piperine influenced the flavours of food items beyond adding a burning or stinging sensation. The results are contradictory as the model solutions and static scales found that piperine enhanced the saltiness perception, but this result was not found during dynamic scaling. Further studies are needed to assess the effects of piperine addition on oral irritation and cross-modal interactions in relation to perceived flavour and textural attributes. Future studies should also investigate how regular consumption of spicy foods influences consumers’ piperine threshold, as well as their perception of foods containing piperine.

## Figures and Tables

**Table 1:** Summary of participants’ demographics.

		Threshold Testing (*n* = 72)	Model Solutions (*n* = 78)	Soup (*n* = 75)
Gender	Male	43	45	42
	Female	57	55	58
Age	18–20	5	6	5
	21–29	30	28	33
	30–39	21	23	23
	40–49	17	14	15
	50–59	17	19	16
	60–65	10	10	8

**Table 2:** Mean (± standard deviation) for the different model solutions containing 0.55 ppm piperine.

	0.55 ppm Piperine	0.2% Xanthan Gum	0.75 mM NaCl	0.55 ppm Piperine and 0.2% Xanthan Gum	0.55 ppm Piperine and 0.75 mM NaCl	0.55 ppm Piperine, 0.75 mM NaCl and 0.2% Xanthan Gum
Saltiness	0.56 a ^1,2,3^ ± 0.07	0.27 b ± 0.02	1.65 c ± 0.15	0.42 ab ± 0.12	1.94 d ± 0.09	1.73 cd ± 0.05
Bitterness	0.64 a ± 0.05	0.31 b ± 0.03	0.50 ab ± 0.08	0.54 ab ± 0.04	0.79 a ± 0.05	0.78 a ± 0.06
Sweetness	0.23 a ± 0.03	0.53 b ± 0.04	0.26 a ± 0.02	0.28 a ± 0.08	0.26 a ± 0.07	0.24 a ± 0.06
Sourness	0.37 a ± 0.04	0.44 a ± 0.06	0.23 a ± 0.05	0.44 a ± 0.05	0.45 a ± 0.03	0.25 a ± 0.02
Umami	0.63 a ± 0.05	0.60 a ± 0.03	0.60 a ± 0.04	0.53 a ± 0.04	0.59 a ± 0.05	0.55 a ± 0.09
Burning and Stinging	1.53 a ± 0.06	0.11 b ± 0.02	0.35 c ± 0.04	1.23 d ± 0.09	1.55 a ± 0.09	1.39 ad ± 0.08

^1^*n* = 78. ^2^ Scores collected on a general labelled magnitude scale where 0 = “No sensation”, and 100 = “Strongest imaginable”, with intermediate descriptors at 1.4, 6, 17, 35, and 51 which are labelled as “barely detectable”, “weak”, “moderate”, “strong”, and “very strong”. ^3^ Means in the same row with the same letter, are not significantly different (*p* > 0.05).

**Table 3:** Consumer mean liking scores (± standard deviation) for appearance, flavour, texture, and overall liking of the different soups with and without piperine (0.55 ppm).

Sample	Appearance	Flavour	Texture	Overall Liking	Saltiness
Control	4.8 a ^1,2,3^ ± 1.1	6.0 a ± 0.9	6.1 a ± 0.8	6.0 a ± 1.1	1.11 a ^4^ ±0.04
Soup with Piperine	5.6 b ± 1.2	5.6 b ± 1.2	6.1 a ± 1.1	5.5 b ± 1.3	1.36 b ± 0.05

^1^ Data input on a 9-point hedonic scale where 1 = Dislike Extremely and 9 = Like Extremely. ^2^ Means in the same column with the same letter (within the same trial) are not significantly different (*p* < 0.05). ^3^ *n* = 100. ^4^ Scores collected on a general labelled magnitude scale where 0 = “No sensation”, and 100 = “Strongest imaginable”, with intermediate descriptors at 1.4, 6, 17, 35, and 51 which are labelled as “barely detectable”, “weak”, “moderate”, “strong”, and “very strong”.

**Table 4:** The average proportion of participants’ citations of TCATA attributes.

Attribute	Control	Soup with Piperine
Salty	0.199 a ^1^	0.160 b
Savoury	0.182 a	0.125 b
Peppery	0.088 a	0.303 b
Sweet	0.132 a	0.049 b
Metallic	0.035 a	0.030 a
Bitter	0.019 a	0.074 b
Sour	0.021 a	0.052 b

^1^ Means in the same row with the same letter, are not significantly different (*p* < 0.05).

## Data Availability

The data presented in this study are available on request from the corresponding author [M.B.M.].
